# Male lake char release taurocholic acid as part of a mating pheromone

**DOI:** 10.1242/jeb.246801

**Published:** 2024-01-25

**Authors:** Tyler J. Buchinger, Ke Li, Ugo Bussy, Belinda Huerta, Sonam Tamrakar, Nicholas S. Johnson, Weiming Li

**Affiliations:** ^1^ Department of Fisheries and Wildlife, Michigan State University, East Lansing, MI 48824, USA; ^2^ US Geological Survey, Great Lakes Science Center, Hammond Bay Biological Station, Millersburg, MI 49759, USA

**Keywords:** Receiver bias, Communication, Chemical signal, Sensory trap

## Abstract

The evolutionary origins of sexual preferences for chemical signals remain poorly understood, due, in part, to scant information on the molecules involved. In the current study, we identified a male pheromone in lake char (*Salvelinus namaycush*) to evaluate the hypothesis that it exploits a non-sexual preference for juvenile odour. In anadromous char species, the odour of stream-resident juveniles guides migratory adults into spawning streams. Lake char are also attracted to juvenile odour but have lost the anadromous phenotype and spawn on nearshore reefs, where juvenile odour does not persist long enough to act as a cue for spawning site selection by adults. Previous behavioural data raised the possibility that males release a pheromone that includes components of juvenile odour. Using metabolomics, we found that the most abundant molecule released by males was also released by juveniles but not females. Tandem mass spectrometry and nuclear magnetic resonance were used to identify the molecule as taurocholic acid (TCA), which was previously implicated as a component of juvenile odour. Additional chemical analyses revealed that males release TCA at high rates via their urine during the spawning season. Finally, picomolar concentrations of TCA attracted pre-spawning and spawning females but not males. Taken together, our results indicate that male lake char release TCA as a mating pheromone and support the hypothesis that the pheromone is a partial match of juvenile odour.

## Teaser

**Summary:** Male lake char (*Salvelinus namaycush*) release a component of juvenile odour to attract females during spawning.

## INTRODUCTION

Some of the most striking exhibits of Earth's biodiversity are sexual signals used by animals to gain access to mates. Unsurprisingly, these traits still captivate evolutionary biologists 150 years after leading Darwin to his idea of sexual selection (Darwin, 1871). In recent years, intensive study of calls, colours and displays has cultivated much theory regarding how preferences for sexual signals evolve. Classic models emphasize the benefits that signals provide to the choosing sex (Andersson and Simmons, 2006) whereas receiver bias models suggest signals exploit pre-existing aspects of receivers' sensory biology (Ryan and Cummings, 2013). Although the goal of these models is to describe common trends across the animal kingdom, studies that provide empirical data have largely focused on a minority of animals and on traits that are conspicuous to humans (Coleman, 2009; Cummings and Endler, 2018; Zuk et al., 2014).

The evolution of sexual preferences remains particularly poorly understood for chemical signals, due, in part, to limited information on the molecules involved (Yohe and Brand, 2018). Chemical traits such as pheromones are the only type of signals likely to be used across the animal Tree of Life because, among other reasons, many animals lack vision and hearing (Buchinger and Li, 2023). Nevertheless, only in insects and a few species of other taxa are the identities of molecules that act as sexual signals known. A possible consequence is that studies on chemically based mate choice have primarily investigated the attributes of signallers (e.g. nutritional, mating or infection status) that correlate with variation in their odours and receivers' preferences for them (Johansson and Jones, 2007), which is a feasible and interesting line of research to pursue without knowing the identity of the compounds. However, this relatively narrow focus on signals that benefit receivers by providing useful information has left receiver bias models little tested as possible evolutionary mechanisms underlying preferences for chemical signals (Yohe and Brand, 2018).

Male lake char, *Salvelinus namaycush* (Walbaum 1792), release a mating pheromone hypothesized to exploit a non-sexual preference for juvenile odour (Buchinger et al., 2015, 2017). Odours of juvenile lake char and its congener Arctic char (*Salvelinus alpinus*) attract conspecific adults (Buchinger et al., 2015; Selset and Døving, 1980). In Arctic char, this attraction is a mechanism by which adults navigate from feeding habitat in the ocean to spawning habitat in streams, where juveniles reside (Nordeng, 1971, 2009). Lake char, however, have lost the migratory phenotype (McLennan, 1994; but see Jones and Ratterman, 2009; Loftus, 1958) and complete their life cycle in lakes where most populations spawn on nearshore reefs several months after juveniles from the previous year-class have left (Deroche, 1969; Martin, 1957). Juvenile odour, although attractive when fresh (Buchinger et al., 2015; Foster, 1985), is unlikely to function as a navigational cue for adults because it does not persist at spawning sites between the time when juveniles emigrate (spring) and adults return to spawn (autumn; Buchinger et al., 2017). Interestingly, male lake char arrive at spawning sites before females (Muir et al., 2012) and release an odour that, like juvenile odour, attracts males and females (Buchinger et al., 2015). Furthermore, males do not behaviourally discriminate between male and juvenile odour and females are attracted to male bile, which contains the bile acids that are likely components of juvenile odour (Buchinger et al., 2020; Zhang et al., 2001). These observations raise the possibility that the non-sexual attraction to juvenile odour generated sexual selection on males to release some of the same molecules as juveniles (i.e. ‘exploit’ the non-sexual attraction to juvenile odour; Christy, 1995).

We sought to identify the male pheromone in lake char to evaluate the hypothesis that it is a partial match of juvenile odour. Whereas previous pheromones in fish were identified using natural product chemistry or screening of commercially available hormones (Li et al., 2018), our prediction that males release a molecule that is present in the odour of juveniles but not females alluded to an alternative approach not yet used to identify fish pheromones: metabolomics (Kuhlisch and Pohnert, 2015). Metabolomics is the global analysis of small molecules in which chemical features, represented by a retention time and mass-to-charge ratio, are compared across groups (Liu and Locasale, 2017). We profiled chemical features of the lake char exometabolome, which consists of the molecules emitted by organisms into the environment and therefore relevant to chemical communication (Izrayelit et al., 2012; Viant et al., 2017), to pinpoint any compounds released by males and juveniles but not females. Using this approach followed by tandem mass spectrometry (MS), nuclear magnetic resonance (NMR), ultra-high performance liquid chromatography–MS/MS (UHPLC-MS/MS) and behavioural assays, we identified taurocholic acid (TCA) – a bile acid previously implicated as a component of juvenile odour (Zhang et al., 2001) – as a male mating pheromone in lake char.

## MATERIALS AND METHODS

### Experimental animals

All experiments were approved by the Michigan State University Animal Use and Care Committee (Animal Use Form Numbers 08/12-148-00, 09/15-135-00, PROTO201800064 and PROTO202100198). Fish were provided by (1) the US Fish and Wildlife Service Sullivan Creek National Fish Hatchery or (2) the US Geological Survey, Great Lakes Science Center, Hammond Bay Biological Station (HBBS), who captured the fish during the spawning season (mid-late October) from a spawning reef in Lake Huron via hook and line. Details of source, strain and ages are provided in the respective experiments below. Experimental animals were stored, sampled and observed at HBBS. Fish from hatcheries were maintained in tanks separated by sex and held at approximately 10°C and a 14 h light:10 h dark photoperiod and then switched to mixed-sex tanks held at 8°C and an 11 h light:13 h dark photoperiod to induce sexual maturation (spermiation and ovulation; Buchinger et al., 2015). All fish were held at ambient Lake Huron temperatures and an 11 h light:13 h dark photoperiod once sexually mature. Fish were given unique tags to pair individual biological data (sex, length, mass) with experimental results and to help track which individuals were used in each experiment. Individuals were implanted with a 23 mm passive integrated transponder tag (PIT tag; Oregon RFID, Portland, OR, USA) via a small incision in the abdomen while briefly immobilized using 0.08% (by volume) clove oil or tagged with unique combinations of up to three streamer tags inserted through the dorsal fin. Adults were fasted throughout the duration of the experiments. All experiments with live animals were done at night as lake char are primarily nocturnal during spawning and, unless otherwise noted, during the autumn (October–December) spawning season (Muir et al., 2012).

### Metabolomic analysis of lake char odours

High resolution-mass spectrometry (HR-MS) on water conditioned by lake char was used to search for putative pheromone components released by males and sexually immature juveniles but not females. Fish used in this experiment were Seneca Lake strain and either age 9–10 years (adults; males: *n*=6, 70.0±5.9 cm, 3.3±1.21 kg; females: *n*=6, 69.7±5.3 cm, 3.17±1.01 kg, means±s.e.m.) or age 4 years (juveniles; *n*=15, 42.5±4.2 cm, 0.7±0.21 kg). Char-conditioned water was collected by placing an individual adult, or 3 juveniles in tanks with 150 l of flow-through ambient-temperature Lake Huron water. Unconditioned Lake Huron water was also collected as a negative control. After a 24 h acclimation of fish to the tank, the flow was shut off and, after 4 h of odour accumulation, 1 liter of water was collected and frozen below −20°C. Later, samples were thawed, and 10 ml was aliquoted and refrozen. The 10 ml subsamples were then freeze-dried using a CentriVap Cold Trap with CentriVap Concentrator (Labconco, Kansas City, MO, USA) and reconstituted in 100 μl of methanol and water (1:1, v:v).

Reconstituted water samples were then subjected to UHPLC coupled to a Xevo G2-S Q-Tof system (Waters Corporation, Milford, MA, USA). Metabolites in reconstituted samples were separated using an ACQUITY C18 BEH UHPLC column (2.1×100 mm, 1.7 μm particle size; Waters Corporation). The column temperature was set at 30°C. The mobile phase consisted of water (A) and acetonitrile (B). The gradient elution was completed using the following gradient program at a flow rate of 250 µl min^–1^ for 10 min: 80% A for 1 min; decreased to 0% A from 1 to 7 min; maintained at 0% A from 7.01 to 9.0 min; back to 80% A from 9.01 min; and maintained to 10 min for column equilibrium. To avoid cross-contamination of samples during the analysis, the needle was washed twice with 80% methanol after each injection. Carry-over from analyte residues was also reduced by injecting 10 μl methanol as a ‘rinsing solution’ on the column after each sample injection using the elution gradients described above.

MS was performed using the negative electrospray ionization mode. For the full-scan MS analysis, spectra were recorded in the range of *m*/*z* 100–1000. Nitrogen gas was used as both the desolvation gas (600 l h^–1^) and cone gas (50 l h^–1^); argon was used as the collision gas at a pressure of 5.3×10^–5^ Torr (0.007 Pa). The source and desolvation temperatures were 100 and 400°C, respectively; the cone voltage and capillary voltage were set to 30 V and 2.8 kV, respectively. The scan time was set at 0.2 s, with an interscan delay of 0.5 s. The LockSpray™ dual electrospray ion source with internal references used for these experiments was leucine enkephalin at a concentration of 100 ng ml^−1^. Lock-mass calibration data at *m*/*z* 554.2615 in negative ion mode were acquired for 1 s every 10 s interval and the flow rate was set at 5 µl min^–1^. For MS/MS, the voltage settings were switched in a quasi-simultaneous fashion to produce non-selective collision-induced dissociation, with collision energies of 5, 10, 20, 30, 40 and 50 eV (Bao et al., 2014).

Kruskal–Wallis rank sum tests followed by pairwise Wilcoxon rank sum tests were used to compare the magnitudes of the top five peaks detected in male-conditioned water with the same five peaks in female, juvenile and control water. No *P*-value adjustment was applied in pairwise Wilcoxon rank sum tests as they were used to explore the data and identify candidate pheromones to be further evaluated in follow-up analyses.

Finally, TCA concentrations in metabolomics samples were quantified using an established method of UHPLC-MS/MS (Li et al., 2015). Reconstituted samples were subjected to UHPLC-MS/MS (Waters Acquity H-class ultra-performance liquid chromatography system; Xevo TQ-S triple mass spectrometer, Waters Corporation) using previously described methods (Li et al., 2015), which include a comparison with authentic TCA. As above, concentrations of TCA (in ng ml^−1^) were compared with Kruskal–Wallis rank sum tests followed by pairwise Wilcoxon rank sum tests with no *P*-value adjustments.

### Isolation of TCA from male urine

To unequivocally confirm the identity of TCA as a component of male odour, we isolated it from male urine and subjected it to NMR. We focused on urine because previous research implicated it as a source of male pheromone in lake char (Buchinger et al., 2020) and we expected urine would be more likely than conditioned water to yield the amount of molecule necessary for NMR. Urine was collected during 2014–2016 from sexually mature male lake char using urinary catheters as previously described (Buchinger et al., 2020). Briefly, males were anaesthetized using clove oil, catheterized with 2 mm tubing inserted into the urinary bladder and secured to the anal, pelvic and dorsal fins, and held in aquaria (∼200 l) supplied with ambient temperature flow-through Lake Huron water. Urine (∼11 l) was collected at least once per day into beakers held on ice, and frozen at <−20°C.

The pooled urine was freeze dried and combined to obtain extract. The extract was then reconstituted in methanol to remove inorganic salts and freeze dried a second time. The final extract (∼500 mg) was dissolved in water and methanol (90:10, v:v) and subjected to a reverse C18 column (house prepared). Fractions (*n*=50) were eluted using an increasing methanol:water gradient, screened using UV-activated thin-layer chromatography (TLC). Similar fractions were combined and the samples concentrated via freeze drying. Samples were then subjected to semi-preparative column chromatography (Luna, 250×10 mm, 5 µm) at ambient temperature. The mobile phase consisted of water (A) and methanol (B). The gradient elution was completed using the following gradient program at a flow rate of 3 ml min^–1^ for 60 min: 90% A for 10 min; decreased to 0% A from 10 to 45 min; and then maintained at 0% A from 45.01 to 60.0 min. The eluents were collected by an autosampler (one fraction per minute). Each fraction was sampled (100 µl) for HR-MS and MS/MS as described above and NMR. An AVANCE 600 MHz instrument (Agilent, Santa Clara, CA, USA) was used to conduct the ^1^H and ^13^C/DEPT-NMR, two-dimensional homonuclear (i.e. COSY) and heteronuclear (i.e. HMQC and HMBC) experiments using previously described parameters (Li et al., 2021).

### TCA release via urine

Urine as a possible route of TCA release was further investigated by sampling wild spermiated male (*n*=7, 71.53±7.48 cm, 3.31±1.16 kg) and ovulated female (*n*=7, 74.61±3.69 cm, 3.38±0.53 kg) lake char. First, individual lake char were acclimated in 150 l of ambient temperature, aerated Lake Huron water for approximately 20 h. The incoming water was shut off several hours prior to sunset and odour was allowed to accumulate for 4 h. A 50 ml water sample was spiked with 10 ng 4-deuterated TCA as an internal standard and frozen at −20°C. The same individuals were then briefly anaesthetized using clove oil, catheterized with 2 mm tubing inserted into the urinary bladder and secured to the anal, pelvic and dorsal fins, and returned to their tank to recover. Approximately 20 h later, urine was collected into a 50 ml vial for 4 h. After 4 h, 1 ml samples of urine were spiked 10 ng 4-deuterated TCA and frozen at −80°C until subsequent analysis using UHPLC-MS/MS (Li et al., 2015). Concentrations (in ng ml^−1^) of TCA in urine and water were compared between males and females using Wilcoxon rank sum tests.

### TCA release rate

Rates of TCA release were estimated for wild males during (November; *n*=12, 74.38±4.79 cm, 3.12±0.66 kg) and outside (April; *n*=12, 73.42±5.09 cm, 2.78±0.59 kg) the spawning season. Males were acclimated in approximately 560 l of ambient Lake Huron water for at least 20 h. Around sunset, the incoming water was shut off and 50 ml water was sampled to quantify the baseline concentration of TCA in the water. After 4 h, a second 50 ml water sample was collected. A set of negative control samples were also collected in April using the same procedure but with no fish added to the tanks (*n*=6). Sub-samples (10 ml) were freeze dried, reconstituted in 1 ml methanol, freeze dried again, reconstituted in 100 μl of 50% methanol:water (v:v), and subjected to UHPLC-MS/MS (Li et al., 2015). The final concentrations (in ng ml^−1^) of TCA after 4 h were compared among spawning season samples, non-spawning season samples and negative control samples using a Kruskal–Wallis rank sum test followed by a pairwise Wilcoxon test with a Benjamini–Hochberg adjustment for multiple comparisons. To estimate release rates, we calculated the difference in TCA concentration between samples collected at the beginning (which included TCA released during acclimation) and end (which included TCA released during acclimation plus TCA released during the experiment) of odour collection.

### Behavioural responses to TCA

**Fig. 1.**
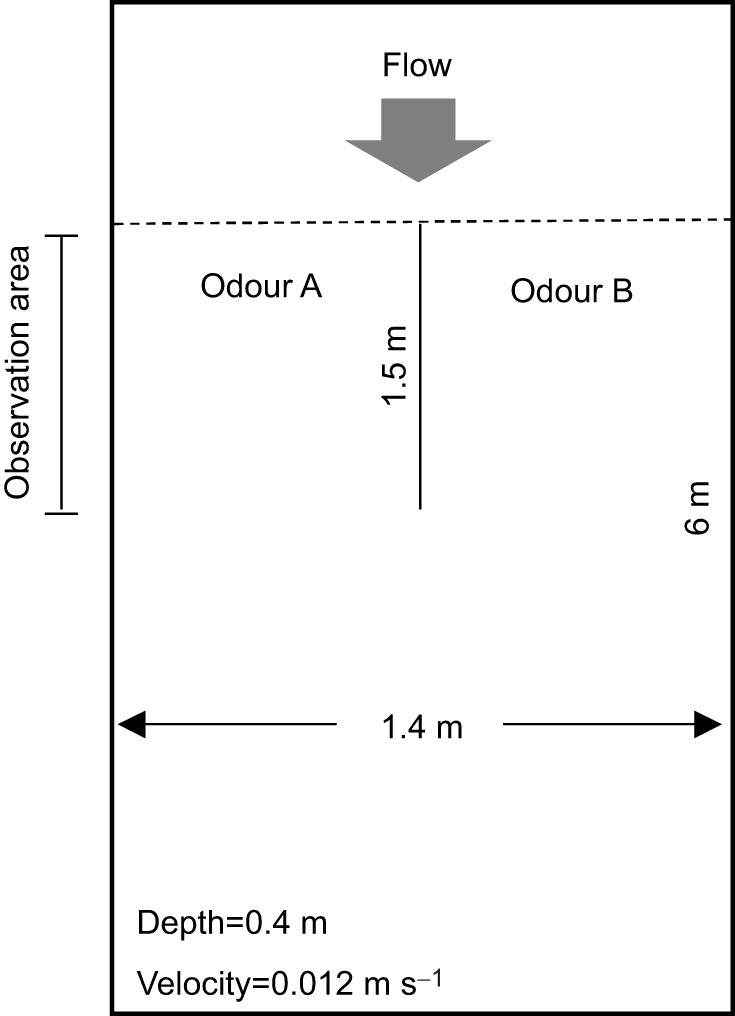
**Behavioural arena used to evaluate responses of adult lake char (*Salvelinus namaycush*) to taurocholic acid (TCA).** Individuals were observed for 30 min before and during application of 1 pmol l^−1^ TCA into one channel (e.g. odour A) and a vehicle control into the second channel (e.g. odour B). Arenas were supplied with Lake Huron water.

Behavioural responses of pre-spawning and spawning male and female lake char to TCA were evaluated using a pair of identical two-choice arenas (see Fig. 1 for dimensions). Synthesized TCA was purchased from Cayman Chemical (www.caymanchem.com; item no. 16215) and a stock solution prepared in methanol and water (1:1, v:v). During trials, TCA was mixed into 9 l of Lake Huron water and applied at 200 ml min^−1^ using a peristaltic pump to reach a concentration of 1 pmol l^−1^ when mixed throughout the full width of the arena. Arenas were built in a flow-through cement flume at the HBBS and supplied with ambient Lake Huron water. A trial began 30 min after sunset when an individual fish was placed in the arena to acclimate. After 30 min, fish were observed for 30 min without any TCA being pumped. TCA was then pumped for a total of 45 min, with a 15 min pre-observation period that allowed the odorant to saturate the activated channel and downstream portion of the arena and a 30 min observation period during which fish were observed. All trials were conducted at night in the dark and the time fish spent in each channel was recorded using infrared cameras. A human observer recorded the time that fish spent in each channel. The observer was not blind to the treatments because of staffing limitations but had no prior expectations as to what responses were predicted. At the conclusion of a trial, the fish was removed, and odours flushed for 15 min before starting the next trial. Dye tests confirmed that 15 min was sufficient to flush the odour from the flume. The R package *lme4* (Bates et al., 2015) was used to construct a linear mixed effect model with a response variable of proportion of time spent in the channel activated with TCA [pTCA=sTCA/(sTCA+sCon); where sTCA is time (s) spent in the channel treated with TCA and sCon is time (s) spent in the channel treated with vehicle control], a fixed effect of period (pre-odour versus odour), and random effect of fish ID. Estimated marginal means and 95% confidence intervals were generated using the *emmeans* package (https://CRAN.R-project.org/package=emmeans) and were compared to 0.5, the expected proportion of time in the activated channel given no response to TCA. Lake char tested for responses to TCA were Seneca Lake strain age 6 males (*n*=25, 59.61±3.49 cm, 2.36±0.38 kg) and age 7 females (*n*=25, 64.06±3.67 cm, 2.75±0.48 kg).

## RESULTS

### Most abundant peak in male exometabolome is also present in that of juveniles but not females

**Fig. 2.**
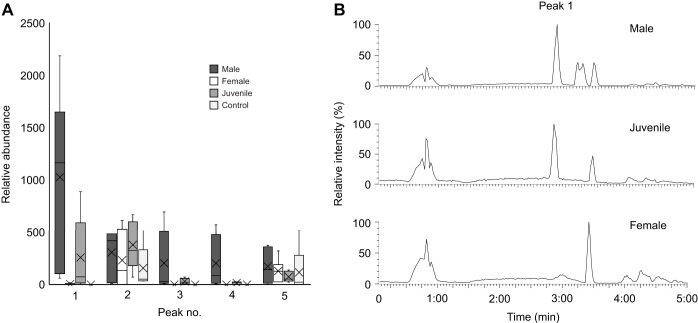
**The most abundant molecule released by male lake char is also released by juveniles but not females.** (A) Metabolomics using high resolution mass spectrometry (HR-MS) revealed the most abundant compound in male odour (peak 1) was the fifth most abundant in juvenile odour but absent from female odour and the negative control. Boxplot (median, upper and lower quartiles and 1.5× interquartile range) shows relative peak areas of the top five compounds detected in water conditioned by males. (B) Representative chromatographs showing peak 1 in water conditioned with male and juvenile but not female lake char. *n*=6 for males and females, *n*=5 for juveniles and control.

Metabolomics using HR-MS revealed the most abundant peak (based on means) in male-conditioned water was the fifth most abundant in juvenile-conditioned water (Fig. 2A). The relative concentrations of peak 1, which had a retention time of 2.85 min (Fig. 2B), were similar in male (*n*=6) versus juvenile (*n*=6) water (Wilcoxon rank sum *P*=0.08), were not different female (*n*=5) versus control (Lake Huron; *n*=5) water (*P*=0.21), and were lower in female and control versus male and juvenile water (*P*<0.05). The second and fifth most abundant peaks in male-conditioned water were at similar relative concentrations across all four groups (male, juvenile, female and control; Kruskal–Wallis *P*>0.05), whereas the third and fourth most abundant peaks were detected at higher relative concentrations in male and juvenile water versus female and control water (*P*<0.05; Fig. 2A). Peaks 3 and 4 were at similar relative concentrations in male versus juvenile water (peak 3 *P*=0.66, peak 4 *P*=0.13), though they tended to be higher in male odour, and in female versus control water (peak 3 *P*=0.56, peak 4 *P*=1). Based upon its *m*/*z* of 514.2853±0.0009 [M−H]^–^ (mean±s.e.m.), male peak 1 was putatively annotated as TCA (Δ=3.77±1.36 ppm).

### MS/MS and NMR confirm male peak 1 is TCA

**Fig. 3.**
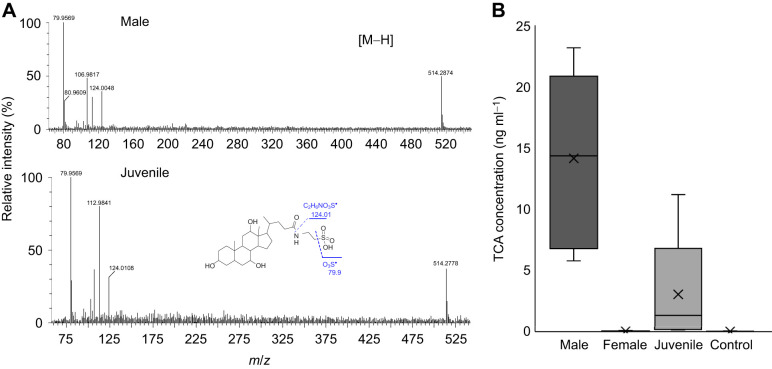
**Peak 1 is taurocholic acid (TCA).** (A) Representative tandem MS (MS/MS) spectra of male- and juvenile-conditioned water showing characteristic ion fragments of TCA (Kaya et al., 2018). (B) Concentrations of TCA in samples used for metabolomics as determined using a previously described method of ultra-high performance liquid chromatography (UHPLC)-MS/MS (Li et al., 2015). *n*=6 for males and females, *n*=5 for juvenile and control.

MS/MS confirmed that peak 1 in male-conditioned water and peak 5 in juvenile-conditioned water were TCA. Electrospray ionization MS under negative ion mode yielded fragments of 79.9 (−SO_3_^–^) and 124.01 (taurine; C_2_H_6_NO_3_S; Fig. 3A), which are characteristic of TCA (Kaya et al., 2018). UHPLC-MS/MS confirmed that TCA was at higher concentrations in male- and juvenile-conditioned water (male 27.39±5.44 nmol l^−1^, juvenile 5.91±4.03 nmol l^−1^) than in female-conditioned water (0.06±0.02 nmol l^−1^) or the Lake Huron controls (0.02±0.02 nmol l^−1^; *P*<0.05; see Fig. 3B for concentrations in ng ml^−1^).

Finally, ^1^H and ^13^C NMR on the residues of urine extract confirmed that TCA was a major chemical constituent of male urine (Table S1, Figs S1–S5).

### Males release TCA at high rates via urine during the spawning season

**Fig. 4.**
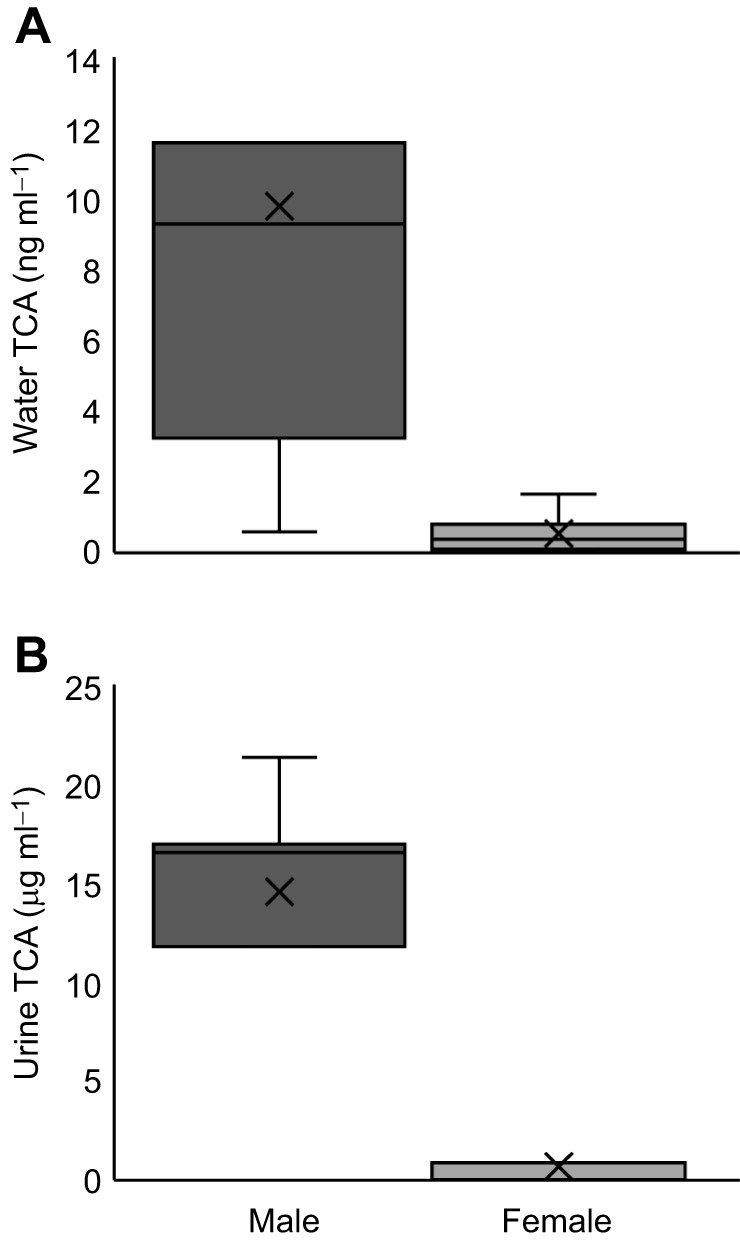
**Male lake char release TCA via urine.** (A) Concentrations of TCA in water conditioned with male and female lake char. (B) Concentrations of TCA in urine collected from male and female lake char. Urine was collected via catheter from spawning males (*n*=7) and females (*n*=7) 24 h after holding water was sampled from the same individuals.

**Fig. 5.**
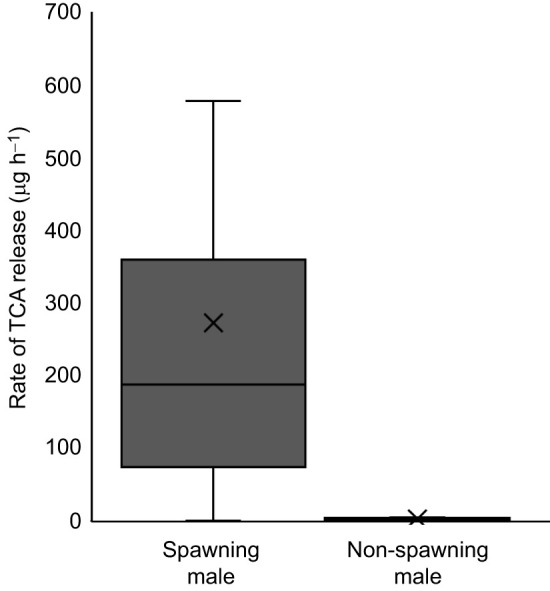
**Male lake char release TCA at high rates during the spawning season.** Release rates of TCA were estimated using paired samples collected at the start (0 h) and end of the odour accumulation period (4 h). Males were sampled during the spawning season (autumn; *n*=12) and outside the spawning season (spring; *n*=11). TCA was detected a low concentrations (0.01±0.001 ng ml^−1^) in the negative control (Lake Huron; *n*=6) samples. For reference, release rates in molar concentrations were 0.53±0.18 µmol h^−1^ for spawning males and 0.008±0.001 µmol h^−1^ for non-spawning males.

Spawning male lake char released TCA via urine at high rates during the spawning season. TCA concentrations were higher in male urine (28.21±4.48 µmol l^−1^; *n*=7) than in female urine (1.35±1.01 µmol l^−1^; *P*=0.001; *n*=7), consistent with the higher concentrations of TCA measured in water conditioned with the same individual males and females 24 h pre-catheterization (male 18.96±6.10 nmol l^−1^, female 1.04±0.42 nmol l^−1^; *P*=0.004; Fig. 4). TCA concentrations were higher in water conditioned by males during versus outside the spawning season (spawning 7.18±3.09 nmol l^−1^, *n*=12; non-spawning 0.07±0.01 nmol l^−1^, *n*=12; *P<*0.001; Fig. 5), though water conditioned by males at either time point had higher TCA concentrations than detected in the negative control (Lake Huron) samples (0.02±0.001 nmol l^−1^; *n*=6; *P<*0.05). Paired samples collected at the start (0 h) and end of the odour accumulation period (4 h) to were used estimate TCA release rates at 76.58±22.66 µg kg^−1^ h^−1^ (0.15±0.04 µmol kg^−1^ h^−1^) for males during the spawning season (*n*=12) and 1.38±0.23 µg kg^−1^ h^−1^ (0.003±0.001 µmol kg^−1^ h^−1^) for males outside the spawning season (*n*=11; see Fig. 5 for release rates unadjusted by body mass). One non-spawning male was excluded from the release rate estimate because its measured TCA concentrations decreased between the 0 h and 4 h sampling periods.

### TCA attracts females but not males

**Fig. 6.**
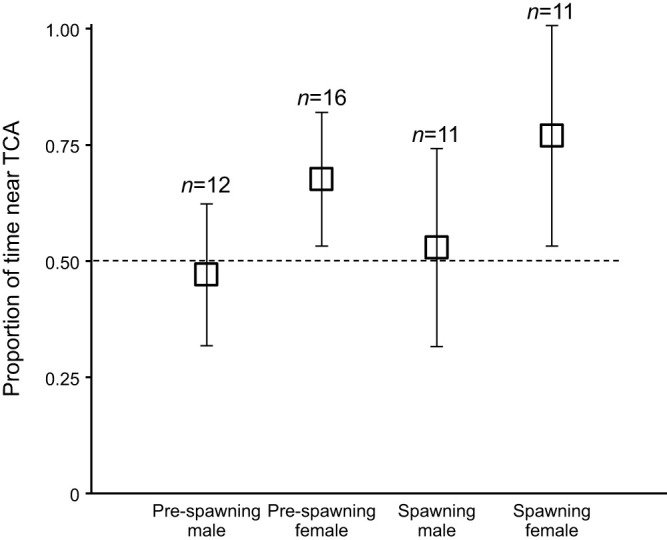
**TCA attracts pre-spawning and spawning female lake char.** Data were analysed as the proportion of time spent in channels treated with TCA versus the vehicle control. Means±95% confidence interval were estimated using a linear mixed effect model with a response variable of the proportion of time spent in the channel activated with TCA, a fixed effect of period (pre-odour versus odour), and a random effect of fish ID. Estimated marginal means and 95% confidence intervals were generated using the *emmeans* package and were compared with 0.5, the expected proportion of time in the activated channel given no response to TCA.

In two-channel flumes, pre-spawning and spawning females but not males spent proportionally more time in the channel activated with 1 pmol l^−1^ TCA than in the channel treated with the vehicle (50% methanol; Fig. 6; 95% confidence interval, CI >0.5, the predicted neutral response). The proportion of time spent in the treatment channel during the pre-odour (negative control) period was not different from 0.5 for any group (pre-spawning male 95% CI: 0.31–0.61; *n*=12, pre-spawning female 95% CI: 0.31–0.60; *n*=16, spawning male 95% CI: 0.26–0.69; *n*=11, spawning female 95% CI: 0.33–0.8; *n*=11). The average time spent in a channel during the 30 min observation periods ranged from 58.73±27.35 s (control channel during odour period for spawning females) to 273.5±62.1 s (control channel during pre-odour period for pre-spawning males). Two trials were excluded from further analysis because the individual did not enter either channel during either the pre-odour or odour periods.

## DISCUSSION

Our results indicate that male lake char release a component of juvenile odour as a mating pheromone. Previous studies have shown that odorants in juvenile faeces may attract adult lake char (Buchinger et al., 2017; Foster, 1985) and closely related Arctic char (Selset and Døving, 1980) to spawning habitat. In the current study, we found that males, but not females, released the bile acid TCA, a common digestive metabolite implicated as a component of juvenile odour (Zhang et al., 2001; Zhang and Hara, 2009; present results). Whereas juveniles release TCA via faeces (Zhang et al., 2001), the primary route of bile acid excretion under normal physiological conditions, males released TCA via urine, which usually has low concentrations of bile acids (Sato and Suzuki, 2001) and is an alternative route of bile acid elimination used (in mammals) during pathological conditions such as cholestasis (Krones et al., 2018). Males released TCA at high rates but only during the spawning season. In laboratory assays, picomolar concentrations of TCA attracted pre-spawning and spawning females but not males. We suggest the non-sexual role of TCA as part of juvenile odour preceded its role as a male pheromone because (1) non-sexual release of TCA via faeces is widespread in vertebrates (Bogevik et al., 2009; Hagey et al., 2010b; Schmucker et al., 2020; Velez et al., 2009; Yeh et al., 2012) and likely an ancestral trait and (2) previous evidence suggests ancestral char use (or used) the odour of juvenile faeces to navigate to spawning sites (Buchinger et al., 2017; Foster, 1985; Nordeng, 1971; Selset and Døving, 1980; Zhang et al., 2001). However, phylogenetic comparisons are needed to further evaluate whether attraction to TCA preceded male signalling with TCA. Nevertheless, our results implicate TCA as a behaviourally active component of juvenile odour and the male mating pheromone, and are consistent with the hypothesis that male signalling with TCA exploits a non-sexual attraction to juvenile odorant.

The few fish pheromones identified using chemistry-driven approaches (Li et al., 2018) challenge conventional models of pheromone evolution that emphasize benefits to receivers (Wisenden, 2015). Many known pheromones in model species such as goldfish are sex steroids and prostaglandins that were commercially available for testing and predicted to act as pheromones because of their roles as hormones that leak into the environment during specific reproductive phases (Stacey, 2015). This biology-driven approach has provided a wealth of information on pheromone function and evolution but risks a bias towards molecules that benefit receivers, as leaked hormones provide information directly related to the reproductive status of potential mates. Indeed, two of the three pheromones previously identified using the alternative approach of isolating molecules from natural odours are non-hormonal compounds (amino acid: Yambe et al., 2006; bile acid: Li et al., 2002) with less obvious links to the reproductive status of the releaser (Buchinger et al., 2014; Li et al., 2018). Our approach leveraged metabolomics as well as natural product chemistry but unveiled a second bile acid mating pheromone. Unlike sex hormones (Stacey, 2015), bile acids are generally not expected to act as mating pheromones because they are primarily involved in digestion of fats, not reproduction (Buchinger et al., 2014). However, in some cases, diet-related differences in bile acid release may indicate reproductive status (Ashouri et al., 2023). Furthermore, adult lake char feed little during the spawning season (Vinson et al., 2021) and the release of large amounts of TCA may require large energy reserves and therefore reflect male quality. Regardless, we expect that additional chemistry-driven studies may continue to reveal unexpected identities of mating pheromones in fish (Li et al., 2018).

The male pheromone in lake char appears to consist of multiple components with sex-specific effects (Buchinger et al., 2015). Pheromones are often mixtures of multiple molecules (Wyatt, 2014) and previous behavioural evidence that females, but not males, discriminate between male and juvenile odour indicates the male lake char pheromone is no exception; males appear to respond to components released by males and juveniles, whereas females respond to components released by males and juveniles plus other components released only by males (Buchinger et al., 2015). In the current study, we searched for and found a component of juvenile odour (TCA) that was released by males at high rates during the spawning season and attracted females. Our experiments did not test the relative role of TCA as a part of a mixture but did show that TCA was the most abundant peak in male-conditioned water. Furthermore, the female responses to TCA we observed were nearly identical to responses to natural male odorant previously observed in a comparable assay (when adjusted for different trial durations; Buchinger et al., 2015). However, TCA at 1 pmol l^−1^ did not attract males, though natural male and juvenile odorants both attract males (Buchinger et al., 2015, 2017) and consist, in part, of TCA (Zhang et al., 2001; present results). Additional components, either alone or as a mixture with TCA, may mediate male responses to male and juvenile odours. Alternatively, males may have higher response thresholds to TCA than females, though, to our knowledge, no data exist on potential sex differences in lake char olfactory responses (but see Ghosal and Sorensen, 2016). Interestingly, sea lamprey (*Petromyzon marinus*) also show sex-specific responses to components of a male pheromone that attracts both sexes (Scott et al., 2019). We postulate that males and females show different responses to chemical constituents of the male pheromone because it plays different ecological roles for each sex (e.g. mate search versus male–male competition; see Buchinger et al., 2015, for more discussion on male responses to male pheromones). More research is needed to better understand the role of TCA as a component of a male pheromone that influences the behaviour of both males and females.

In conclusion, our study provides evidence that male lake char release TCA as a mating pheromone that exploits a non-sexual preference for juvenile odour. Our chemistry-driven approach implicated an evolutionary mechanism inconsistent with conventional models of mating pheromones in fish (Stacey, 2015; Wisenden, 2015) and with little empirical support in even the well-characterized pheromones of insects (Stökl and Steiger, 2017). Hypothesis-driven metabolomics proved to be a powerful approach that may accelerate and enrich research on pheromone communication in fish and other taxa for which identification of underlying chemical traits is often logistically prohibitive. As TCA is a common bile acid produced by many animals (Hagey et al., 2010a; Hofmann et al., 2010), future work might also evaluate whether and how the male pheromone in lake char is species specific, which is an often noted, but not required, characteristic of pheromones (Buchinger and Li, 2020). Notably, both the current study and previous work support suggestions that spawning lake char also use cues detected by other sensory modalities (e.g. hearing: Johnson et al., 2018) as females spent only about 10% of the trial in the channel treated with natural male odorant (Buchinger et al., 2015) or TCA (current study). Other pheromone components and sensory modalities may allow species-specific communication despite TCA being a common metabolite. Lastly, a better understanding of pheromone communication in lake char may inform management of both native and invasive populations (Hansen et al., 2019).

## Supplementary Material

10.1242/jexbio.246801_sup1 Supplementary information

## References

[JEB246801C1] Andersson, M. and Simmons, L. W. (2006). Sexual selection and mate choice. *Trends Ecol. Evol.* 21, 296-302. 10.1016/j.tree.2006.03.015 16769428

[JEB246801C2] Ashouri, S., Da Silva, J. P., Canário, A. V. and Hubbard, P. C. (2023). Bile acids as putative social signals in Mozambique tilapia (*Oreochromis mossambicus*). *Physiol. Behav.* 272, 114378. 10.1016/j.physbeh.2023.114378 37858914

[JEB246801C3] Bao, J., Gao, X. and Jones, A. D. (2014). Unusual negative charge-directed fragmentation: collision-induced dissociation of cyclopentenone oxylipins in negative ion mode. *Rap. Comm. Mass Spectrom* 28, 457-464. 10.1002/rcm.6803 24497283

[JEB246801C4] Bates, D., Mächler, M., Bolker, B. and Walker, S. (2015). Fitting linear mixed-effects models using lme4. *J. Stat. Softw.* 67, 1-48. 10.18637/jss.v067.i01

[JEB246801C5] Bogevik, A. S., Tocher, D. R., Langmyhr, E., Waagbø, R. and Olsen, R. E. (2009). Atlantic salmon (*Salmo salar*) postsmolts adapt lipid digestion according to elevated dietary wax esters from Calanus finmarchicus. *Aquac. Nutr.* 15, 94-103. 10.1111/j.1365-2095.2008.00571.x

[JEB246801C6] Buchinger, T. J. and Li, W. (2020). The evolution of (non) species-specific pheromones. *Evol. Ecol.* 34, 455-468. 10.1007/s10682-020-10046-0

[JEB246801C7] Buchinger, T. J. and Li, W. (2023). Chemical communication and its role in sexual selection across Animalia. *Commun. Biol.* 6, 1178. 37985853 10.1038/s42003-023-05572-w PMC10662023

[JEB246801C8] Buchinger, T. J., Li, W. and Johnson, N. S. (2014). Bile salts as semiochemicals in fish. *Chem. Senses* 39, 647-654. 10.1093/chemse/bju039 25151152

[JEB246801C9] Buchinger, T. J., Li, W. and Johnson, N. S. (2015). Behavioral evidence for a role of chemoreception during reproduction in lake trout. *Can. J. Fish. Aquat. Sci.* 72, 1847-1852. 10.1139/cjfas-2015-0351

[JEB246801C10] Buchinger, T. J., Marsden, J. E., Binder, T. R., Huertas, M., Bussy, U., Li, K., Hanson, J. E., Krueger, C. C., Li, W. and Johnson, N. S. (2017). Temporal constraints on the potential role of fry odors as cues of past reproductive success for spawning lake trout. *Ecol. Evol.* 7, 10196-10206. 10.1002/ece3.3546 29238547 PMC5723602

[JEB246801C11] Buchinger, T. J., Li, W. and Johnson, N. S. (2020). Behavioural responses of female lake trout *Salvelinus namaycush* to male chemical stimuli and prostaglandin F2α. *J. Fish Biol.* 97, 1224-1227. 10.1111/jfb.14446 32592402

[JEB246801C12] Buchinger, T. J. et al. (2024). Male lake char release taurocholic acid as part of a mimetic pheromone. Dryad Dataset. 10.5061/dryad.8931zcrvk PMC10906664 38270203

[JEB246801C13] Christy, J. H. (1995). Mimicry, mate choice, and the sensory trap hypothesis. *Am. Nat.* 146, 171-181. 10.1086/285793

[JEB246801C14] Coleman, S. W. (2009). Taxonomic and sensory biases in the mate-choice literature: there are far too few studies of chemical and multimodal communication. *Acta Ethol.* 12, 45-48. 10.1007/s10211-008-0050-5

[JEB246801C15] Cummings, M. E. and Endler, J. A. (2018). 25 Years of sensory drive: the evidence and its watery bias. *Curr. Zool.* 64, 471-484. 10.1093/cz/zoy043 30108628 PMC6084598

[JEB246801C16] Darwin, C. (1871). *The Descent of Man, and Selection in Relation to Sex*. Princeton University Press.

[JEB246801C17] Deroche, S. E. (1969). Observations on the spawning habits and early life of lake trout. *Prog. Fish. Cult.* 31, 109-113. 10.1577/1548-8640(1969)31[109:OOTSHA]2.0.CO;2

[JEB246801C18] Foster, N. R. (1985). Lake trout reproductive behavior: influence of chemosensory cues from young-of-the-year by-products. *Trans. Am. Fish. Soc.* 114, 794-803. 10.1577/1548-8659(1985)114<794:LTRB>2.0.CO;2

[JEB246801C19] Ghosal, R. and Sorensen, P. W. (2016). Male-typical courtship, spawning behavior, and olfactory sensitivity are induced to different extents by androgens in the goldfish suggesting they are controlled by different neuroendocrine mechanisms. *Gen. Comp. Endocrinol.* 232, 160-173. 10.1016/j.ygcen.2016.04.028 27131392

[JEB246801C20] Hagey, L. R., Møller, P. R., Hofmann, A. F. and Krasowski, M. D. (2010a). Diversity of bile salts in fish and amphibians: evolution of a complex biochemical pathway. *Physiol. Biochem. Zool.* 83, 308-321. 10.1086/649966 20113173 PMC2845723

[JEB246801C21] Hagey, L. R., Vidal, N., Hofmann, A. F. and Krasowski, M. D. (2010b). Evolutionary diversity of bile salts in reptiles and mammals, including analysis of ancient human and extinct giant ground sloth coprolites. *BMC Evol. Biol.* 10, 1-23. 10.1186/1471-2148-10-133 20444292 PMC2886068

[JEB246801C22] Hansen, M. J., Guy, C. S., Budy, P. and McMahon, T. E. (2019). Trout as native and nonnative species: a management paradox. In *Trout and Char of the World* (ed. J. Kershner, J. Williams, R. Gresswell and J. Lobon-Cervia), pp. 645-684. American Fishery Society.

[JEB246801C23] Hofmann, A. F., Hagey, L. R. and Krasowski, M. D. (2010). Bile salts of vertebrates: structural variation and possible evolutionary significance. *J. Lipid. Res.* 51, 226-246. 10.1194/jlr.R000042 19638645 PMC2803226

[JEB246801C24] Izrayelit, Y., Srinivasan, J., Campbell, S. L., Jo, Y., von Reuss, S. H., Genoff, M. C., Sternberg, P. W. and Schroeder, F. C. (2012). Targeted metabolomics reveals a male pheromone and sex-specific ascaroside biosynthesis in Caenorhabditis elegans. *ACS Chem. Biol.* 7, 1321-1325. 10.1021/cb300169c 22662967 PMC3423530

[JEB246801C25] Johansson, B. G. and Jones, T. M. (2007). The role of chemical communication in mate choice. *Biol. Rev. Camb. Philos. Soc.* 82, 265-289. 10.1111/j.1469-185X.2007.00009.x 17437561

[JEB246801C26] Johnson, N. S., Higgs, D., Binder, T. R., Marsden, J. E., Buchinger, T., Brege, L., Bruning, T., Farha, S. and Krueger, C. C. (2018). Evidence of sound production by spawning lake trout (*Salvelinus namaycush*) in lakes Huron and Champlain. *Can. J. Fish. Aquat. Sci.* 75, 429-438. 10.1139/cjfas-2016-0511

[JEB246801C27] Jones, A. G. and Ratterman, N. L. (2009). Mate choice and sexual selection: what have we learned since Darwin? *Proc. Natl. Acad. Sci. USA* 106, 10001-10008. 10.1073/pnas.0901129106 19528643 PMC2702796

[JEB246801C28] Kaya, I., Jennische, E., Lange, S. and Malmberg, P. (2018). Dual polarity MALDI imaging mass spectrometry on the same pixel points reveals spatial lipid localizations at high-spatial resolutions in rat small intestine. *Anal. Methods* 10, 2428-2435. 10.1039/C8AY00645H 31490465 PMC5985652

[JEB246801C29] Krones, E., Pollheimer, M. J., Rosenkranz, A. R. and Fickert, P. (2018). Cholemic nephropathy–historical notes and novel perspectives. *Biochim. Biophys. Acta Mol. Basis Dis.* 1864, 1356-1366. 10.1016/j.bbadis.2017.08.028 28851656

[JEB246801C30] Kuhlisch, C. and Pohnert, G. (2015). Metabolomics in chemical ecology. *Nat. Prod. Rep.* 32, 937-955. 10.1039/C5NP00003C 25926134

[JEB246801C31] Li, K., Buchinger, T. J., Bussy, U., Fissette, S. D., Johnson, N. S. and Li, W. (2015). Quantification of 15 bile acids in lake charr feces by ultra-high performance liquid chromatography–tandem mass spectrometry. *J. Chromatogr. B Analyt. Technol. Biomed. Life Sci.* 1001, 27-34. 10.1016/j.jchromb.2015.07.028 26253808

[JEB246801C32] Li, K., Buchinger, T. J. and Li, W. (2018). Discovery and characterization of natural products that act as pheromones in fish. *Nat. Prod. Rep.* 35, 501-513. 10.1039/C8NP00003D 29662986

[JEB246801C33] Li, K., Siefkes, M. J. and Li, W. (2021). Cervidins AD: novel glycine conjugated fatty acids from the tarsal gland of male whitetail deer, *Odocoileus virginianus*. *J. Chem. Ecol.* 47, 243-247. 10.1007/s10886-021-01255-0 33629151

[JEB246801C34] Li, W., Scott, A. P., Siefkes, M. J., Yan, H., Liu, Q., Yun, S.-S. and Gage, D. A. (2002). Bile acid secreted by male sea lamprey that acts as a sex pheromone. *Science* 296, 138-141. 10.1126/science.1067797 11935026

[JEB246801C35] Liu, X. and Locasale, J. W. (2017). Metabolomics: a primer. *Trends Biochem. Sci.* 42, 274-284. 10.1016/j.tibs.2017.01.004 28196646 PMC5376220

[JEB246801C36] Loftus, K. (1958). Studies on river-spawning populations of lake trout in eastern Lake Superior. *Trans. Am. Fish. Soc.* 87, 259-277. 10.1577/1548-8659(1957)87[259:SORPOL]2.0.CO;2

[JEB246801C37] Martin, N. V. (1957). Reproduction of lake trout in Algonquin Park, Ontario. *Trans. Am. Fish. Soc.* 86, 231-244. 10.1577/1548-8659(1956)86[231:ROLTIA]2.0.CO;2

[JEB246801C38] McLennan, D. A. (1994). A phylogenetic approach to the evolution of fish behaviour. *Rev. Fish Biol Fish.* 4, 430-460. 10.1007/BF00042889

[JEB246801C39] Muir, A., Blackie, C., Marsden, J. and Krueger, C. (2012). Lake charr *Salvelinus namaycush* spawning behaviour: new field observations and a review of current knowledge. *Rev. Fish Biol. Fish.* 22, 575-593. 10.1007/s11160-012-9258-6

[JEB246801C40] Nordeng, H. (1971). Is the local orientation of anadromous fishes determined by pheromones? *Nature* 233, 411-413. 10.1038/233411a0 16063406

[JEB246801C41] Nordeng, H. (2009). Char ecology. Natal homing in sympatric populations of anadromous Arctic char *Salvelinus alpinus* (L.): roles of pheromone recognition. *Ecol. Freshw. Fish.* 18, 41-51. 10.1111/j.1600-0633.2008.00320.x

[JEB246801C42] Ryan, M. J. and Cummings, M. E. (2013). Perceptual biases and mate choice. *Annu. Rev. Ecol. Evol. Syst.* 44, 437-459. 10.1146/annurev-ecolsys-110512-135901

[JEB246801C43] Sato, K. and Suzuki, N. (2001). Whole-cell response characteristics of ciliated and microvillous olfactory receptor neurons to amino acids, pheromone candidates and urine in rainbow trout. *Chem. Senses* 26, 1145-1156. 10.1093/chemse/26.9.1145 11705800

[JEB246801C44] Schmucker, A. K., Johnson, N. S., Bussy, U., Li, K., Galbraith, H. S., Chung-Davidson, Y. W. and Li, W. (2020). American eels produce and release bile acid profiles that vary across life stage. *J. Fish Biol.* 96, 1024-1033. 10.1111/jfb.14295 32072638

[JEB246801C45] Scott, A. M., Zhang, Z., Jia, L., Li, K., Zhang, Q., Dexheimer, T., Ellsworth, E., Ren, J., Chung-Davidson, Y.-W. and Zu, Y. (2019). Spermine in semen of male sea lamprey acts as a sex pheromone. *PLoS Biol.* 17, e3000332. 10.1371/journal.pbio.3000332 31287811 PMC6615597

[JEB246801C46] Selset, R. and Døving, K. B. (1980). Behaviour of mature anadromous char (*Salmo alpinus* L.) towards odorants produced by smolts of their own population. *Acta Physiol. Scand* 108, 113-122. 10.1111/j.1748-1716.1980.tb06508.x 7376909

[JEB246801C47] Stacey, N. (2015). Hormonally derived pheromones in teleost fishes. In *Fish Pheromones and Related Cues* (ed. P. W. Sorensen and B. D. Wisenden), pp. 33-88. John Wiley & Sons, Inc.

[JEB246801C48] Stökl, J. and Steiger, S. (2017). Evolutionary origin of insect pheromones. *Curr. Opin. Insect Sci.* 24, 36-42. 10.1016/j.cois.2017.09.004 29208221

[JEB246801C49] Velez, Z., Hubbard, P. C., Welham, K., Hardege, J. D., Barata, E. N. and Canário, A. V. (2009). Identification, release and olfactory detection of bile salts in the intestinal fluid of the Senegalese sole (Solea senegalensis). *J. Comp. Physiol. A Neuroethol. Sens. Neural Behav. Physiol.* 195, 691-698. 10.1007/s00359-009-0444-5 19415298

[JEB246801C50] Viant, M. R., Elphinstone Davis, J., Duffy, C., Engel, J., Stenton, C., Sebire, M. and Katsiadaki, I. (2017). Application of passive sampling to characterise the fish exometabolome. *Metabolites* 7, 8. 10.3390/metabo7010008 28216558 PMC5372211

[JEB246801C51] Vinson, M. R., Chavarie, L., Rosinski, C. L. and Swanson, H. K. (2021). Trophic ecology. In *The Lake Charr Salvelinus namaycush: Biology, Ecology, Distribution, and Management* (ed. A. Muir, C. Krueger, M. J. Hansen and S. C. Riley), pp. 287-314: Springer.

[JEB246801C52] Wisenden, B. D. (2015). The cue–signal continuum: a hypothesized evolutionary trajectory for chemical communication in fishes. In *Fish Pheromones and Related Cues* (ed. P. W. Sorensen and B. D. Wisenden), pp. 149-158. John Wiley & Sons, Inc.

[JEB246801C53] Wyatt, T. D. (2014). *Pheromones and Animal Behavior: Chemical Signals and Signatures*: Cambridge University Press.

[JEB246801C54] Yambe, H., Kitamura, S., Kamio, M., Yamada, M., Matsunaga, S., Fusetani, N. and Yamazaki, F. (2006). L-Kynurenine, an amino acid identified as a sex pheromone in the urine of ovulated female masu salmon. *Proc. Natl. Acad. Sci. USA* 103, 15370-15374. 10.1073/pnas.0604340103 17030810 PMC1622830

[JEB246801C55] Yeh, C.-Y., Chung-Davidson, Y.-W., Wang, H., Li, K. and Li, W. (2012). Intestinal synthesis and secretion of bile salts as an adaptation to developmental biliary atresia in the sea lamprey. *Proc. Natl. Acad. Sci. USA* 109, 11419-11424. 10.1073/pnas.1203008109 22733776 PMC3396466

[JEB246801C56] Yohe, L. R. and Brand, P. (2018). Evolutionary ecology of chemosensation and its role in sensory drive. *Curr. Zool.* 64, 525-533. 10.1093/cz/zoy048 30108633 PMC6084603

[JEB246801C57] Zhang, C. and Hara, T. J. (2009). Lake char (*Salvelinus namaycush*) olfactory neurons are highly sensitive and specific to bile acids. *J. Comp. Physiol. A Neuroethol. Sens. Neural. Behav. Physiol.* 195, 203-215. 10.1007/s00359-008-0399-y 19137319

[JEB246801C58] Zhang, C., Brown, S. B. and Hara, T. J. (2001). Biochemical and physiological evidence that bile acids produced and released by lake char (*Salvelinus namaycush*) function as chemical signals. *J. Comp. Physiol. B Biochem. Syst. Environ. Physiol.* 171, 161-171. 10.1007/s003600000170 11302533

[JEB246801C59] Zuk, M., Garcia-Gonzalez, F., Herberstein, M. E. and Simmons, L. W. (2014). Model systems, taxonomic bias, and sexual selection: beyond *Drosophila*. *Annu. Rev. Entomol.* 59, 321-338. 10.1146/annurev-ento-011613-162014 24160422

