# Supplementary material for: Male lake char release taurocholic acid as part of a mating pheromone
Source: J Exp Biol. 2024 Jan 25;227(2):jeb246801. doi: 10.1242/jeb.246801 (PMC10906664; doi:10.1242/jeb.246801)
Supplement: Supplementary information [file jexbio-227-246801-s1.pdf]

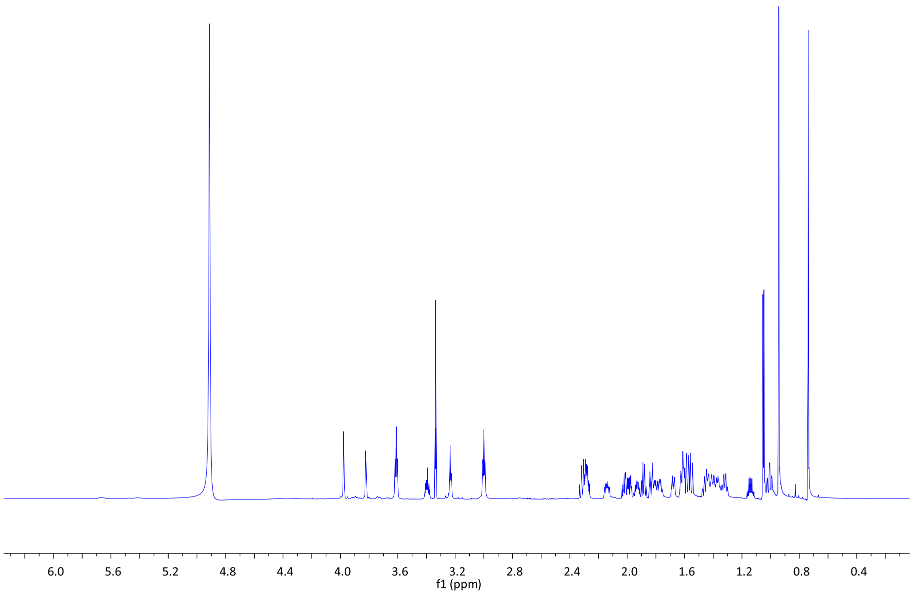

Fig. S1. ^{1}H nuclear magnetic resonance (NMR) spectrum of taurocholic acid (TCA) isolated from male lake char (*Salvelinus namaycush*) urine in methanol-*d*$_4$.

Journal of Experimental Biology • Supplementary information

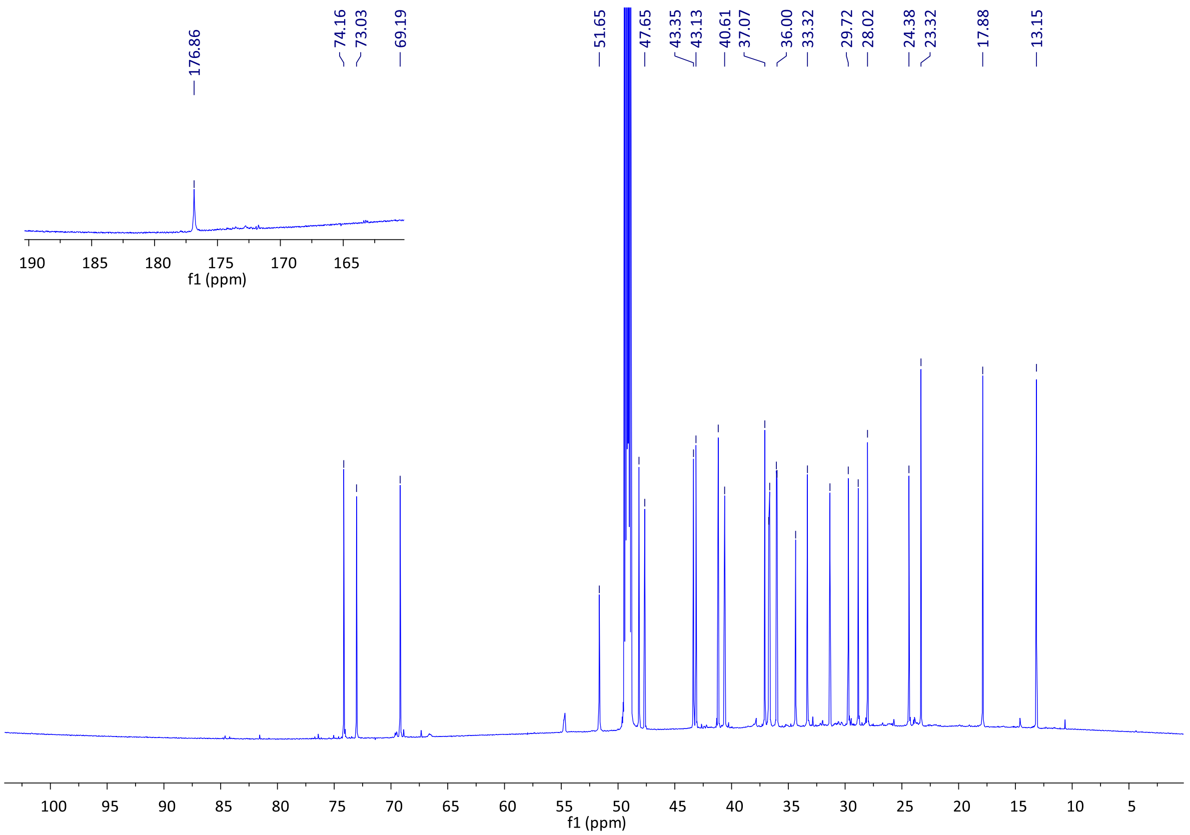

Fig. S2. ^{13}C nuclear magnetic resonance (NMR) spectra of taurocholic acid (TCA) isolated from male lake char (*Salvelinus namaycush*) urine in methanol-*d*$_4$.

Journal of Experimental Biology • Supplementary information

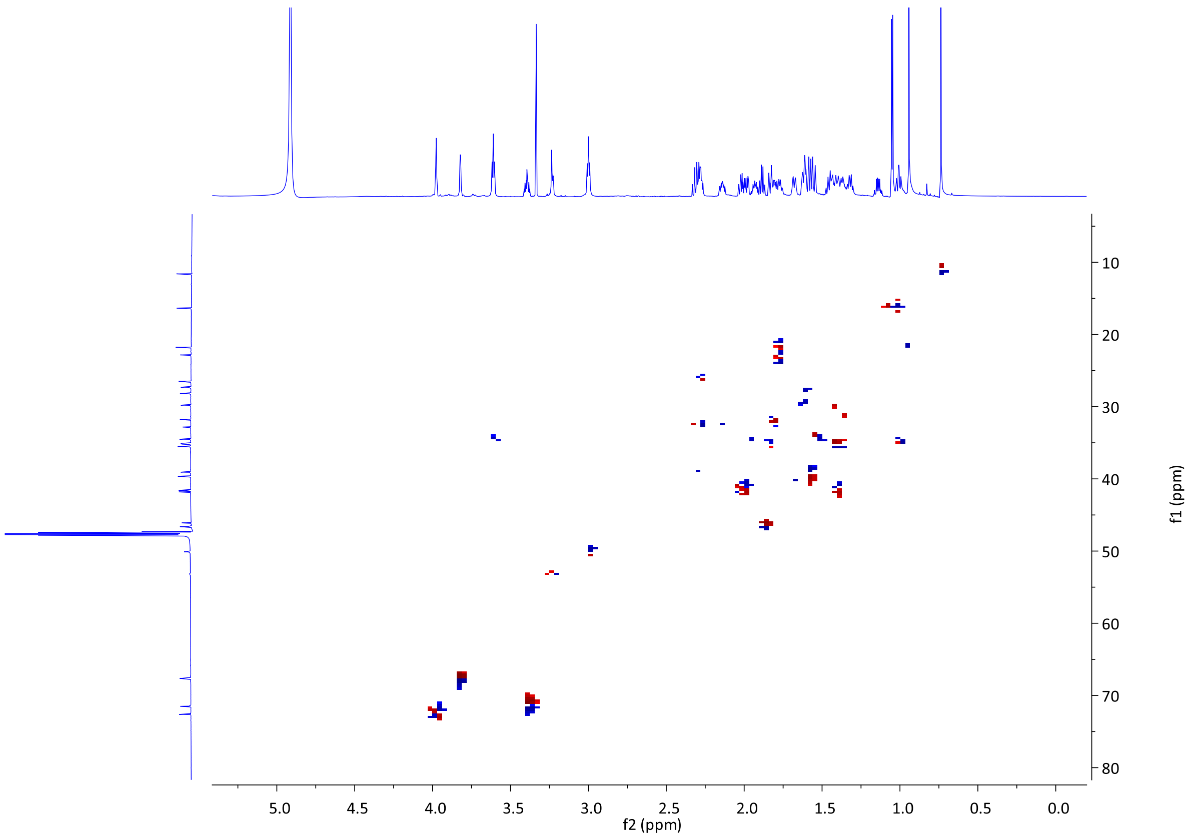

Fig. S3. Heteronuclear single quantum coherence (HSQC) spectrum of taurocholic acid (TCA) isolated from male lake char (*Salvelinus namaycush*) urine in methanol-d_4.

Journal of Experimental Biology • Supplementary information

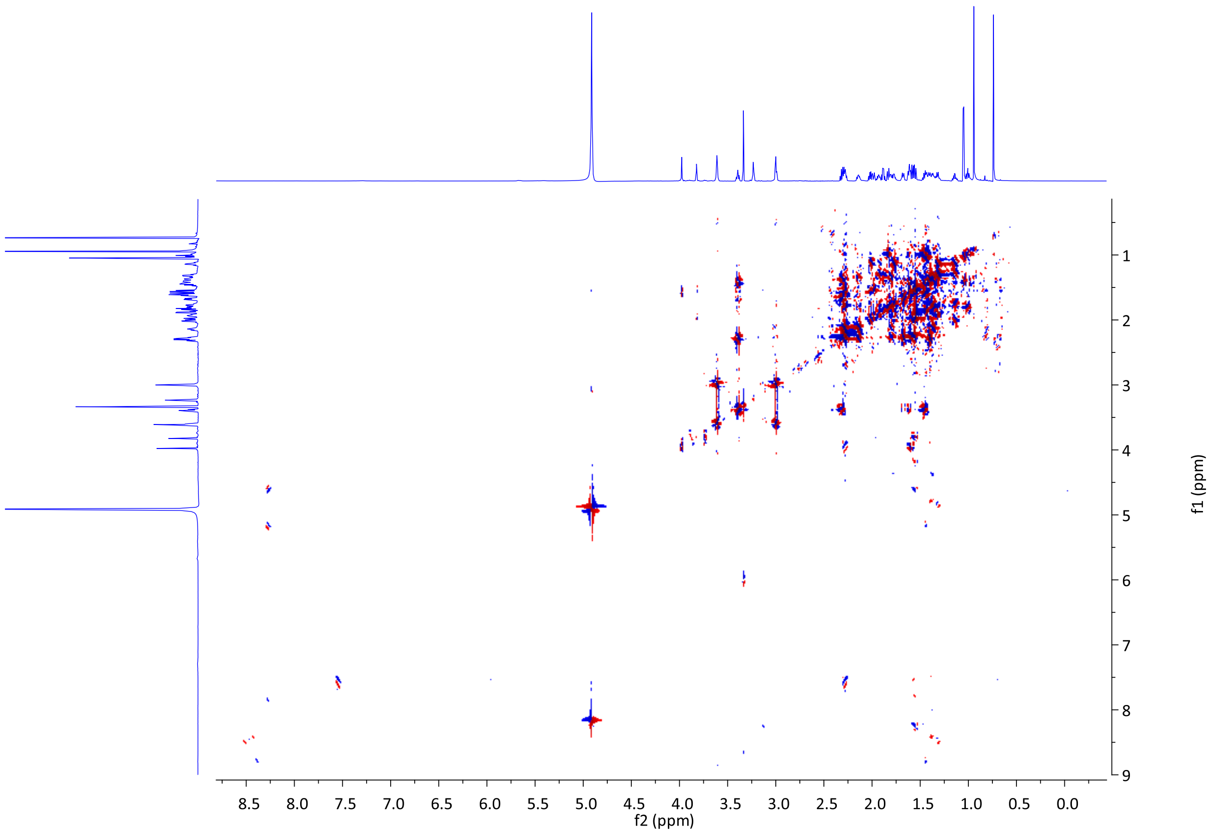

Fig. S4. ^{1}H-^{1}H correlated spectroscopy (COSY) spectrum of taurocholic acid (TCA) isolated from male lake char (*Salvelinus namaycush*) urine in methanol-*d*$_4$.

Journal of Experimental Biology • Supplementary information

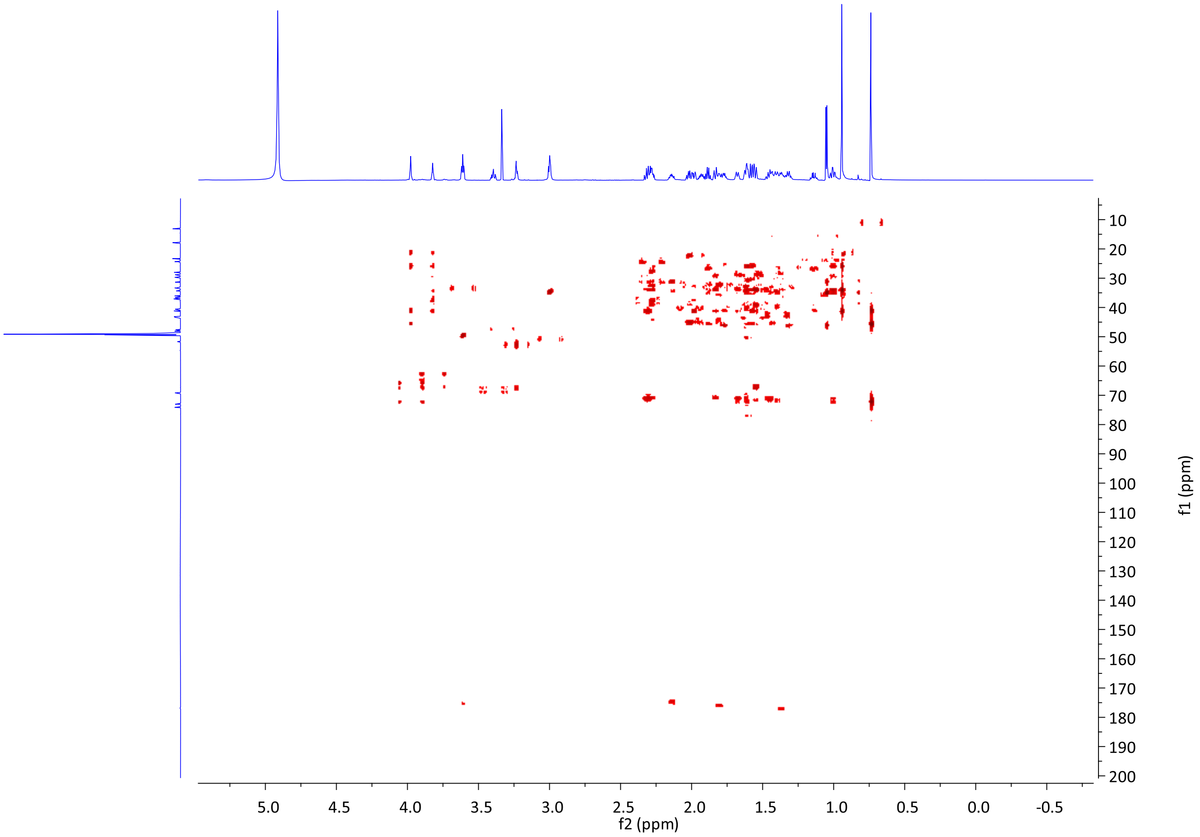

Fig. S5. Heteronuclear multiple bond correlation (HMBC) spectrum of taurocholic acid (TCA) isolated from male lake char (*Salvelinus namaycush*) urine in methanol-d_4.

Journal of Experimental Biology • Supplementary information

Table S1. ^{13}C Chemical shifts of taurocholic acid (TCA) in methanol-d_4

No.	δ_C	No.	δ_C
1	36.73 (CH_2)	14	43.35 (CH)
2	31.33 (CH_2)	15	24.38 (CH_2)
3	73.03 (CH)	16	28.85 (CH_2)
4	40.61 (CH_2)	17	48.15 (CH)
5	43.30 (CH)	18	13.15 (CH_3)
6	36.00 (CH_2)	19	23.32 (CH_3)
7	69.19 (CH)	20	37.07 (CH)
8	41.17 (CH)	21	17.88 (CH_3)
9	28.02 (CH)	22	33.32 (CH_2)
10	36.64 (qC)	23	34.36 (CH_2)
11	29.72 (CH_2)	24	176.86 (qC)
12	74.16 (CH)	25	36.05 (CH_2)
13	47.65 (qC)	26	51.65 (CH_2)

Journal of Experimental Biology • Supplementary information
